# Iowa State Dental Society

**Published:** 1867-08

**Authors:** 


### PROCEEDINGS OF THE IOWA STATE DENTAL SOCIETY.

*Fifth Annual Meeting, held at Lyons, July* 9, 10, *and* 11, '67.

### FIRST DAY.

LYONS, Tuesday, July 9—8 p. m.

The Iowa State Dental Society met in the Randall House parlor, the President, Dr. Kulp, in the chair; Dr. Chase, Secretary *pro tem.*

On motion a committee to nominate officers for the ensuing year, consisting of Drs. Chase, McGarvey and Sanborn.

Dr. Wilson, of Boonsboro, and Dr. Thomas, of Muscatine, were elected members of the Society.

Drs. Sanborn, Kulp and McGarvey were appointed a Committee on Examination of Candidates.

The Committee on Incorporation, through Dr. Chase, reported that this Society was now incorporated. Report accepted and committee discharged.

Dr. Sayles was appointed a committee to procure a reporter for the session.

The following resolutions were offered by Dr. Chase, and adopted:

*Resolved*, That we, as members of the Iowa State Dental Society, hereby pledge our honor that we will hereafter require nothing short of two years pupilage of those who shall come under our instruction, and graduation in some regular Dental College, as sufficient preparatory study for entrance into the Dental profession.

*Resolved*, That the facilities of Dental education are now so great that there is no valid excuse for the student for non-attendance on the lectures of our Dental Colleges.

*Resolved*, That we hereby offer our hearty support to the Dental Colleges of this country, which are so laudably endeavoring to raise the standard of professional education.

The subject of Salivary Calculus was discussed by Drs. Sanborn and Chase.

Adjourned till Wednesday morning at 8 o'clock.

SECOND DAY.

Wednesday, July, 10—8 a. m.

Society organized, with the President, Dr. W. O. Kulp, in the chair; Dr. J. Hardman, Secretary.

Dr. J. H. Nicholson, of Anamosa, was elected to membership in the Society.

On motion of Dr. Myers, certain names of Dentists, who from absence or other cause fail to attend the meetings of the Society, were ordered to be stricken from the roll.

Dr. Sanborn, chairman of committee on order of business, reported a programme, which was adopted.

The President announced that Dr. Sayles, resident Dentist, invited the Association to his house on Thursday evening, to meet the medical profession of Lyons and Clinton for social intercourse.

Dr. Smith read an essay on "Dental Quackery," and a discussion followed on the subject, participated in by Drs. Chase, Tulloss and Wilson, and Prof. Peebles, of St. Louis.

On motion of Dr. Chase, the committee on legislation was instructed to draft a bill conforming with the bill before the Ohio Legislature, and to furnish dentists with copies of the same, with petitions for its passage—the petitions with signatures to be returned to the chairman of the committee before the meeting of the next Legislature.

Drs. Ingersoll, Chase and Tulloss were designated as gentlemen to be recommended to the Governor for a Board of Examiners, in case the proposed law was enacted.

Dr. Kulp offered the following resolution, which was unanimously adopted:

*Resolved*, That we, as a Society, hereby endorse the Missouri Dental College in the effort to make our profession what it truly is—a specialty of medicine.

Dr. Sanborn read an essay on "The Relation of Matter," and Drs. Chase, Ingersoll, Smith and Hardman further discussed the subject.

The Treasurer's report was read and accepted.

Dr. Sanborn read an essay on "Cellular Physiology," illustrating his remarks by diagrams.

On motion of Dr. Sayles, the medical profession of Lyons were invited to participate with the Society in its discussions.

Adjourned to 1½ p. m.

Wednesday, July 10—1½ p. m.

Upon assembling, an election of officers for the ensuing year was had, resulting as follows:

President—Dr. J. F. Sanborn, of Tabor, Fremont Co.

Vice President—Dr. A. B. Mason, of Waterloo.

Corresponding Secretary—Dr. H. S. Chase, of Iowa City.

Recording Secretary and Treasurer—Dr. Wilson of Boonsboro.

On taking the chair, the President thanked the Society for the honor conferred upon him; and the retiring officer read an address containing the history of the association.

Dr. Kulp read an essay on "The Principles of Plugging Teeth," and a discussion ensued, in which Drs. Ingersoll, Chase, Tulloss, Kulp and Prof. Peebles, engaged.

Dr. Hardman read an address on "Anæsthesia," after which discussion was had between Drs. Tulloss, Ingersoll and Chase.

Dr. Hudson, resident physician of Lyons, by request, read a paper upon the subject, "Anæsthesia," followed by remarks by Drs. Hardman, Wilson and Chase.

The thanks of the Society were voted Dr. Hudson for his treatise.

Prof. Peebles, of the Missouri Dental College, of St. Louis, was invited to speak, and upon compliance with the request, received a vote of thanks.

Drs. Sanborn, Chase and Myers were appointed a committee to select a curriculum for the proposed Dental Department of the Keokuk Medical College, and to designate a gentleman for the Professorship.

The Corresponding Secretary was instructed to open correspondence with the State Medical Society, and to invite the sending of a delegate to this Society's next meeting.

An hour was passed in the report of cases and examination of instruments, etc., and the Association adjourned to 8 o'clock.

Wednesday, July 10—8 p. m.

Society met, with Vice-President in the chair.

The subject of "Alveolar Hemorrhage" was treated by Dr. Chase in a short paper, and discussed by Drs. Hardman, Ingersoll, Hudson, Smith, Severance, Wilson, Chase, Tulloss, Mason, Kulp, Sayles, Bronson and Gunkel, and Prof. Judd, nearly all citing cases of their own in illustration of their views. Dr. Lothrop, resident physician of Lyons, also made some remarks upon the treatment of hemorrhage.

Dr. Ingersoll read a report or treatise on "Dental Nomenclature," which led to a discussion, in which Drs. Wilson, Ingersoll, Chase and Kulp and Prof. Judd, participated.

Adjourned till 8 o'clock Thursday morning.

THIRD DAY.

Thursday, July 11—8 a. m.

Society met, Dr. Kulp, in the President's absence, in the chair.

Dr. Mason reported verbally on the "Making and Tempering of Instruments," and the subject was further discussed by Drs. Smith, Hardman, Severance and Poor.

Keokuk was selected as the place for the holding of the next meeting of the Society.

The Society resolved upon the establishment of a Professorship of the "Principles of Dental Science," in the Keokuk

Medical School, and Dr. Ingersoll was chosen as the occupant of the chair, and empowered to call such assistance from the Society as he required. The pecuniary arrangements were left for the consideration of the college faculty and Dr. Ingersoll.

An amendment to the constitution, providing for the admission of Dental students as junior members, was adopted.

A letter was read from Dr. Cochran, excusing himself for non-attendance.

The subject of inflammation was discussed—Prof. Judd, and Drs. Ingersoll, Kulp, Chase and Hardman speaking. Drs. Massman and Lyons, of this city, also made some remarks on the question.

Dr. Chase read a paper upon "Peri-cementitis," and the subject was further dwelt upon by Drs. Kulp, Magill, Wilson, Severance, Smith, Bronson, Sayles and Mason, and by Dr. Hudson and Prof. Judd.

Adjourned to 1½ p. m.

Thursday, July 11—1½ p. m.

Society met, the President in the chair.

Dr. R. S. Rathbun, of McGregor, was elected a junior member of the Society.

The subject of "Treatment of the Dental Pulp" was discussed by Drs. Chase, Wilson, Ingersoll, Sanborn, Tulloss and Kulp, and Prof. Peebles.

On the "Pathology of Dental Decay," Prof. Judd and Drs. Sanborn and Chase held a brief debate.

On "Sensitive Dentine," Drs. Ingersoll, Chase, Kulp, Tulloss and Sanborn, and Profs. Judd and Peebles made some remarks.

On the subject of "Alveolar Abscess," Drs. Kulp, Sanborn, Chase and Bronson, and Prof. Judd, each made some remarks.

Dr. Kulp was elected a delegate to the Chicago Dental Society, to meet on the 12th inst.

Dr. Ingersoll was elected representative to the Missouri Convention; Dr. Bronson alternate.

Dr. Chase was elected representative to attend the Illinois Convention; Dr. Sayles, alternate.

Drs. Tulloss, Poor, Severance, Smith, Mason, Thomas and Wilson were elected delegates to the American Dental Association, to meet at Cincinnati; any members present at such meeting, not named, to act as delegates.

The Corresponding Secretary was instructed to assign subjects to members for essays at the next year's session.

The Corresponding Secretary was instructed to send a copy of the proceedings to a Dental journal for publication.

Prof. Judd was elected a corresponding member of the Society.

Dr. Chase was continued as Committee on Hygiene.

Thanks were tendered to Dr. Chase and son (who drew the diagrams exhibited;) to Profs. Peebles and Judd for their attendance and instructions; to the Northern Line Packet Company; to the Proprietor of the Randall House; and to the reporter for his faithfulness.

The support of the Society was pledged to Mr. J. H. Canon, of Muscatine, for the establishment of a Dental depot in the State.

Adjourned *sine die*.

